# A Measurement-Supported Extrapolation Framework for Lowband MIMO Coverage and Capacity Enhancement in Future AAS-Assisted Wireless Networks

**DOI:** 10.3390/s26134297

**Published:** 2026-07-06

**Authors:** Kornél Merkli, Szilvia Nagy, Péter Prukner

**Affiliations:** 1 Digital Development Center, Széchenyi István University, 9026 Győr, Hungary; peter.prukner@ddc.sze.hu; 2 Faculty of Informatics and Electrical Engineering, Széchenyi István University, 9026 Győr, Hungary; nagysz@sze.hu

**Keywords:** lowband active antenna systems, lowband MIMO, coverage and capacity enhancement, cell-edge throughput sustainability, AAS-assisted beamforming, midband and highband offloading

## Abstract

Low-frequency mobile bands remain essential for wide-area and penetration-limited wireless coverage, but their limited channel bandwidth constrains the achievable capacity. This paper presents a measurement-supported extrapolation framework for assessing how lowband MIMO and future AAS-assisted operation can enhance coverage and single-user throughput-oriented capacity in wireless networks. The motivation is to evaluate whether such deployments can strengthen the lower-frequency layer as a robust coverage-and-capacity support layer for general traffic and reduce the load on midband and higher-frequency resources. Controlled radiated SISO and 2×2 MIMO measurements were performed with a base-station simulator and commercial user equipment in representative lowband and midband frequency bands. Measured RSRP, CQI, BLER, MAC-layer throughput, and IP-layer throughput thresholds for a 25 Mbit/s downlink target were used for coverage estimation and conditional extrapolation. Under the Extended Hata model, the measured 2×2 MIMO thresholds yielded a 43% larger estimated radius at 800 MHz than at 1800 MHz, while the same model indicated a 93% radius increase for a representative 10 dB AAS-related beamforming gain scenario. Conditional 4×4 MIMO extrapolations indicated data rates above 100 Mbit/s in 10 MHz and above 200 Mbit/s with 10 MHz two-component-carrier aggregation under ideal high-CQI conditions. The results support the potential of future lowband AAS deployments. The AAS and higher-order MIMO results are scenario-based estimates rather than direct field validation.

## 1. Introduction

Reliable wide-area coverage remains a fundamental requirement of fifth-generation (5G) and beyond cellular networks, particularly in rural, suburban, and indoor environments, where large service areas must be maintained with a limited number of base-station sites. While midband 5G deployments can provide high peak data rates through wider channel bandwidths, their higher path loss and less favorable diffraction and penetration characteristics may limit the achievable throughput at the cell edge, particularly under coverage-limited conditions [1,2,3,4]. Low-frequency mobile bands, especially the 700–900 MHz range, therefore remain essential coverage layers due to their favorable propagation characteristics, robust indoor penetration, and improved link reliability under challenging radio conditions. The principal limitation of lowband operation is the restricted amount of available spectrum. Consequently, capacity enhancement in these bands must rely on improved spectral efficiency, link robustness, and spatial reuse rather than on bandwidth expansion. Conventional lowband deployments typically employ passive sector antennas and support a limited number of spatial layers, most commonly 2×2 multiple-input multiple-output (MIMO). Active antenna systems (AAS), by contrast, enable more flexible control of the radiated field through element- or subarray-level excitation, allowing beamforming gains, improved spatial selectivity, and more efficient interference management. When combined with MIMO transmission, these capabilities can enhance the received signal quality and increase the throughput-oriented service capabilities of the lowband coverage layer [5,6,7].

Large-scale AAS and massive MIMO solutions are currently predominantly deployed in midband and higher-frequency 5G systems, particularly around 3.5 GHz and in millimeter-wave bands, where beam management, highly directional transmission, and multi-stream operation are central aspects [8,9,10]. However, recent industrial and research developments indicate the gradual extension of active antenna architectures toward lower Frequency Range 1 (FR1) bands, including 1.8–2.6 GHz and, in the longer term, the 700–900 MHz spectrum range [11,12,13,14,15]. In these lower-frequency bands, the main expected benefit is not the allocation of a large number of spatial layers to a single user equipment device, as practical terminal capabilities remain limited. Instead, AAS-assisted operation is expected to provide moderate beamforming gains, support for higher-order MIMO such as 4×4 MIMO, improved spatial discrimination, and more efficient multi-user resource utilization. In this role, it should not be viewed as a direct competitor to wide midband or higher-frequency allocations in terms of peak capacity. Rather, it can strengthen the lower-frequency layer as a stable coverage-and-capacity support layer for general users, moderate-throughput services, rural coverage, and penetration-limited scenarios. By serving such traffic more reliably in this layer, part of the general traffic load can be shifted away from midband and higher-frequency resources. This allows these resources to be reserved for applications requiring larger bandwidths, higher peak rates, or lower latency, or those with stricter quality-of-service requirements, including critical service categories.

The objective of this study is to develop and apply a throughput-driven measurement and extrapolation framework for evaluating the roles of MIMO links and AAS-assisted operation in future lowband mobile network deployments. The motivation is to assess whether such deployments can strengthen the coverage layer as a stable coverage-and-capacity support layer for moderate-throughput services, penetration-limited users, and general traffic offloading from midband and higher-frequency resources. Controlled laboratory measurements were performed with a base-station simulator, a commercial user equipment device, and radiated single-input single-output (SISO) and 2×2 MIMO links in representative lowband and midband mobile frequency bands. The measurements determine the reference signal received power (RSRP), channel quality indicator (CQI), block error rate (BLER), MAC-layer throughput, and IP-layer throughput conditions required to sustain a fixed 25 Mbit/s downlink service target under limited channel bandwidth (BW) values of 5–10 MHz [4].

The main contribution of this work is a measurement-based framework that uses the measured 2×2 MIMO thresholds as a common basis for lowband–midband coverage comparison and for future lowband AAS scenario evaluation. Coverage is estimated with both a Friis upper-bound reference and an empirical Extended Hata model. The measured link behavior is then extended through an AAS-related beamforming gain scenario, a conditional 4×4 MIMO coverage and capacity extrapolation, and a 4×4 MIMO throughput projection with carrier aggregation using Third Generation Partnership Project (3GPP) transport block size relationships. The framework therefore supports the assessment of the usable service area, cell-edge throughput stability, and moderate-rate traffic support, while clearly separating the measured results from extrapolated AAS-assisted and higher-order MIMO scenarios. In this study, capacity is interpreted in a throughput-oriented sense, referring to the measured and extrapolated downlink data rate capability under the considered bandwidth, carrier aggregation, and MIMO assumptions. The potential midband and highband offloading role is therefore treated as a qualitative network-level implication of improved lowband service robustness, rather than as a directly measured multi-user or scheduler-level result.

The present study focuses on controlled link-level measurements and conditional extrapolations, rather than on the full system-level validation of a deployed lowband AAS or massive MIMO network. The base-station simulator and horn antennas were used to establish a reproducible radiated link-level measurement platform, while the semi-anechoic chamber ensured measurement isolation and repeatability. However, this environment cannot represent the full complexity of outdoor cellular propagation. Therefore, the measured results should be interpreted as controlled performance thresholds, whereas the AAS-assisted and higher-order MIMO results should be interpreted as scenario-based estimates. Owing to the structural similarities between Long-Term Evolution (LTE) and 5G New Radio (5G NR) physical-layer operation in FR1 bands, the observed link-level trends are relevant for future lowband 5G-oriented studies, particularly for 5G NR measurement methodology and preliminary coverage assessment studies [16,17]. However, the RSRP-to-throughput relationship is not directly transferable to 5G NR without validation, because NR uses flexible numerology; different reference-signal structures, such as synchronization signal blocks and CSI-RS, instead of LTE cell-specific reference signals; and different CQI and link adaptation configurations. Therefore, direct validation in 5G NR remains necessary. These aspects are identified as future work, including direct 5G NR validation, lowband 4×4 MIMO testing, representative AAS radiation patterns, and outdoor multi-user investigations.

The article is organized as follows. Section 2 reviews lowband MIMO, active antenna systems, and capacity enhancement techniques relevant to wide-area cellular coverage. Section 3 presents the proposed measurement and extrapolation methodology, including the equipment, configuration, propagation models, and throughput threshold evaluation procedure. Section 4 discusses the measured results and the corresponding AAS and higher-order MIMO extrapolations. Finally, Section 5 summarizes the main conclusions and outlines future research directions.

## 2. State of the Art in Lowband MIMO and AAS-Assisted Performance Enhancement

Lowband coverage enhancement has been widely studied in the context of rural and sparsely populated areas, where conventional site densification is often economically impractical. A comprehensive survey of 5G coverage improvement techniques [18] highlights the roles of advanced antenna architectures, heterogeneous deployment strategies, and adaptive radio-resource management in such environments. Experimental evidence from the Agriculture and Rural Communities (ARA) wireless living laboratory [19] further shows that multi-band, MIMO-enabled programmable radio architectures can sustain high-capacity links over multi-kilometer distances while reducing rural deployment costs. Related studies on large-scale multi-user MIMO (MU-MIMO) arrays installed on television towers [20,21] demonstrate that high-elevation infrastructure combined with large antenna apertures can substantially improve rural coverage and uplink channel quality under constrained equivalent isotropically radiated power (EIRP) conditions. Beyond terrestrial macro-cell deployments, rural coverage has also been investigated in the context of fixed wireless access (FWA) and hybrid terrestrial/non-terrestrial architectures. Network planning studies for rural FWA [22] show that MU-MIMO and block-based precoding can increase the number of simultaneously served households and support the identification of capacity and density thresholds for economically viable deployment. Comparative analyses of terrestrial network (TN) and non-terrestrial network (NTN) architectures [23] further suggest that hybrid satellite–terrestrial solutions may provide additional flexibility in deep-rural scenarios where conventional terrestrial infrastructure alone may be insufficient.

Several studies have investigated how AAS-based beamforming can improve FR1 cellular performance compared with conventional passive sector antennas. Field investigations of AAS capabilities [24] show that horizontal and vertical beamforming, electrical tilt adaptation, and spatially selective coverage shaping can provide substantial capacity improvements and enhance coverage uniformity in wide-area deployments. Complementary singular value decomposition (SVD)-based beamforming vector optimization [25] indicates that optimized precoding can yield cell-edge signal-to-interference-plus-noise ratio (SINR) improvements on the order of 8–10 dB under suitable channel conditions. System-level evaluations of 5G beam management [26] further show that beam search, tracking, and update mechanisms directly affect the achievable performance, particularly in networks with large cell footprints, where delayed or inaccurate beam adaptation may degrade link quality. These studies collectively indicate that AAS and beamforming can provide meaningful improvements over passive sectorized lowband deployments. Nevertheless, the realized gain in practical networks remains scenario-dependent and is influenced by the propagation environment, antenna radiation pattern, user angular distribution, precoding strategy, and interference conditions.

Massive MIMO and large-scale AAS are currently more mature in midband and higher-frequency 5G deployments than in the lowband spectrum. Nevertheless, developments in modular massive MIMO architectures [27] indicate that large antenna apertures can be decomposed into flexible subarray structures while retaining the beamforming and spatial processing advantages of larger arrays. Such modularity is relevant for future lowband systems, where the physical aperture size, tower loading, deployment costs, and terminal-layer limitations impose practical constraints on antenna design. The impact of MIMO order and precoding has also been examined in several studies. Investigations of massive MIMO and linear precoding techniques [28,29] show that zero-forcing (ZF) and minimum mean-square error (MMSE) precoding can reduce interference and improve spectral efficiency when the channel supports spatial multiplexing. These studies indicate that 4×4 MIMO can provide a meaningful gain over 2×2 MIMO under favorable propagation and channel-rank conditions. At the same time, empirical measurements in reverberation chambers [30] demonstrate that the realized throughput gain is affected by channel correlation and fading dynamics: 2×2 configurations may exhibit stronger performance variability, whereas 4×4 MIMO can provide more stable throughput when sufficient decorrelation is present. These findings highlight that higher-order MIMO gains should not be treated as deterministic scaling factors, especially at the lowband cell edge. This consideration is reflected in the conditional interpretation and non-ideal sensitivity discussion of the 4×4 MIMO projections in this study.

The reviewed literature shows that lowband coverage enhancement requires the combined consideration of propagation, the antenna architecture, the MIMO order, beamforming, and network-level deployment constraints. Prior work has addressed rural coverage improvement techniques [18], programmable and MIMO-enabled rural testbeds [19], high-tower MU-MIMO architectures [20,21], modular massive MIMO concepts [27], rural FWA planning [22], hybrid TN/NTN coverage solutions [23], AAS field performance [24], beamforming vector optimization [25], beam management mechanisms [26], massive MIMO precoding [28,29], and chamber-based MIMO performance evaluation [30]. However, only a limited number of studies have employed controlled throughput measurements to link lowband and midband performance thresholds with future AAS and higher-order MIMO scenarios. The novelty of this work lies not in the radiated 2×2 MIMO measurement procedure itself—which is related to established multi-antenna testing methodologies such as 3GPP TR 37.977—but in the use of measured throughput thresholds as empirical anchors for lowband to midband comparison, as well as for the conditional extrapolation to AAS-assisted and higher-order MIMO scenarios. The present work addresses this gap by using SISO and 2×2 MIMO measurements as the empirical basis for estimating how an AAS-related beamforming gain scenario, 4×4 MIMO, and carrier aggregation could improve the coverage and service stability of the lowband layer. Accordingly, the extrapolated results are treated as measurement-supported scenario estimates for future lowband AAS operation.

## 3. Measurement and Extrapolation Method for Assessing Lowband MIMO- and AAS-Assisted Coverage and Capacity Enhancement

The measurements were conducted in representative lowband and midband FR1 mobile frequency ranges. In the implemented base-station simulator configuration, the investigated carriers corresponded to Band 20, Band 3, and Band 1, denoted in this study as L08, L18, and L21, respectively. These bands were selected to compare low-frequency coverage behavior with midband operation under identical throughput-oriented evaluation criteria and to provide a measurement basis for future lowband AAS-oriented extrapolations.

### 3.1. Measurement Equipment

The measuring instruments and auxiliary equipment used for the controlled lowband and midband measurements are listed in Table 1.

### 3.2. Measurement Setup

The measurements were carried out in a semi-anechoic chamber (SAC), a shielded radio-frequency (RF) test environment designed for controlled and repeatable radiated measurements. The dedicated feedthrough connectors integrated into the chamber enabled uninterrupted signal transmission from the base-station simulator to the antennas positioned inside the chamber. The SAC provided sufficient isolation from external mobile networks and ensured that the conducted tests did not introduce interference into operational licensed networks. Due to its partially reflective structure, the SAC can introduce limited and repeatable reflected components that support the observation of radiated MIMO link behavior under controlled laboratory conditions. At the same time, the SAC environment should not be interpreted as a complete representation of outdoor cellular propagation, since it does not reproduce effects such as diffraction, large-scale shadowing, mobility-induced fading, or multi-cell interference [35]. Therefore, the measured results are interpreted as controlled link-level performance thresholds rather than as direct real-world performance validation. The measurement configuration represents a single-cell, single-UE downlink link-level scenario.

Within this controlled radiated setup, the horn antennas were used as passive transmitting antennas to establish stable and repeatable SISO and 2×2 MIMO links. For MIMO operation, whether based on spatial multiplexing or diversity, sufficiently low correlation between the propagation paths is required. A commonly used practical design rule is to apply antenna–element spacing of at least 0.5–1 λ [36]. In the present radiated setup, the aperture characteristics and directivity of the horn antennas required larger separation to obtain stable stream distinguishability at the receiver. Therefore, the final configuration used inter-antenna spacing of about 1.72 λ in each investigated frequency band. The wavelengths and corresponding physical antenna spacings associated with the downlink center frequencies are summarized in Table 2. It should be noted that the dual-horn arrangement was selected to provide a stable and repeatable radiated two-stream laboratory link, not to reproduce the polarization structure of a deployed lowband sector antenna. Practical lowband 2×2 deployments commonly employ cross-polarized antenna elements, whereas the present setup primarily relies on spatial separation and chamber realization to support stream distinguishability. Therefore, the measured 2×2 MIMO baseline should be interpreted as a controlled link-level reference rather than as a direct representation of a specific cross-polarized macro-cell deployment. This limitation is considered when interpreting the higher-order MIMO extrapolations.

The transmitter–receiver separation was also a critical design parameter. It had to be short enough to preserve distinguishability between the streams at the receiver, while being sufficiently large to avoid dominant near-field effects. In the initial validation phase, the first condition was verified by confirming that the maximum available data rate could be achieved at the maximum transmit power in all examined frequency bands. To address the second condition, the plane-wave model (PWM) and spherical-wave model (SWM) were compared for the present measurement geometry to determine the minimum distance at which the phase error caused by wavefront curvature becomes negligible [37]. This model comparison was used only for configuring the chamber measurement distance. It is separate from the cell radius estimation step, where the Friis and Extended Hata propagation models described in Section 3.3 are applied. Due to the symmetry of the setup and the small spacing of the user equipment (UE) antenna elements, the associated path-length variations were sufficiently small for amplitude variations to be neglected.

#### Determination of the Optimal Measuring Distance

The plane-wave model considers only a single line-of-sight (LOS) component, resulting in a planar waveform. In this case, the points of equal phase form a plane, and, if the incoming wavefront forms an angle with a uniform linear antenna array (ULA), the phase difference between the individual elements can be derived from basic geometric considerations and is given by the following equation:

(1) $$\Delta \Phi =\frac{2\pi }{\lambda }d_{r}\cos\left(\theta_{r}\right),$$

where λ [m] is the wavelength of the radio signal, $d_{r}$ [m] is the spacing between the elements of the ULA, and $\theta_{r}$ [rad] is the angle between the line defined by the ULA at the receiver and the plane of the incoming wavefront [37].

In contrast, the spherical-wave model accounts for the exact path lengths between antenna elements, as expressed in (2), allowing the identification of multiple independent modes at short distances [37]:

(2) $$H_{m,n}=\exp\left(j\frac{2\pi r_{m,n}}{\lambda }\right),$$

where $H_{m,n}$ is the channel matrix element of a MIMO system with *M* transmit and *N* receive antennas, $r_{m,n}$ [m] is the path length between the indexed transmit and receive antenna elements, and λ [m] is the wavelength of the radio signal.

Following the methodology presented in [37,38], the discrepancy between the two models was characterized by examining the square of the singular values of the channel matrix H, which correspond to the eigenvalues of the matrix

(3) $$W=\left\{\begin{array}{cc} HH^{H}, & M<N,\\ H^{H}H, & M\geq N, \end{array}\right.$$

where *M* and *N* are the numbers of transmit and receive antennas, respectively; H is the channel matrix; and $H^{H}$ is the Hermitian transpose of H. Under the plane-wave model, the channel matrix has rank 1 and therefore the dominant eigenvalue is given in the form $\rho_{U,V}^{P}$. In the SWM case, the eigenvalues also depend on the geometric parameters, resulting in the presence of multiple dominant modes at short transmitter–receiver distances [37].

To quantify the accuracy of the approximation, the parameter g∈[0,1) was introduced. As defined in (4), this parameter relates the largest eigenvalues of the channel matrices associated with the two propagation models,

(4) $$\rho_{U,V}^{S}\leq g\cdot \rho_{U,V}^{P}$$

where $\rho_{U,V}^{S}$ and $\rho_{U,V}^{P}$ are the largest eigenvalues of the channel matrix for the SWM and PWM models, respectively, and *g* is the ratio of these eigenvalues.

The PWM is considered sufficiently accurate if the contribution of the additional spherical-wave modes remains small enough that the capacity predicted by the SWM does not exceed the PWM-based capacity by more than the prescribed tolerance. This tolerance is expressed by the capacity ratio κ between the SWM and PWM descriptions. Following the derivation in [37,38], the admissible upper bound of the eigenvalue ratio *g* is obtained by imposing this capacity ratio constraint for a given number of transmit antennas *M* and average SNR $\bar{\gamma }$. Thus, the inequality below is not introduced as a new propagation model but as a model selection criterion that limits the allowable SWM-PWM capacity deviation:

(5) $$g\leq \frac{1}{2}+\frac{1}{2}\sqrt{1-\frac{4}{{\left(M\bar{\gamma }\right)}^{2}}\left(1+M\bar{\gamma }\right)\left({\left(1+M\bar{\gamma }\right)}^{\kappa -1}-1\right)},$$

where *g* is the maximum eigenvalue ratio of the channel matrix for the PWM and SWM, *M* is the number of transmitter antennas, $\bar{\gamma }$ [dB] is the average signal-to-noise ratio of the measurement setup, and κ is the theoretical capacity ratio derived from the PWM and SWM [37].

The value of κ was set to 1.05, corresponding to a maximum 5% capacity deviation between the PWM and SWM descriptions. The average SNR was chosen as 20 dB, which is representative of high-channel-quality conditions corresponding to CQI = 15 [39]. The geometric dependency enters the formulation through the parameter τ, as given in (6):

(6) $$\tau =\frac{\lambda R}{d_{t}d_{r}\cos\theta_{t}\cos\theta_{r}},$$

where *R* [m] is the distance between the axes of the transmit and receive antennas; $d_{t}$ [m] and $d_{r}$ [m] are the element spacings of the transmit and receive arrays, respectively; and $\theta_{t}$ [rad] and $\theta_{r}$ [rad] are the angles between the plane defined by the incoming wavefront and the ULA axis at the transmitter and receiver, respectively.

For the 2 × 2 case, the decision threshold can be expressed in closed form [38], from which the physical distance threshold $R_{g}$ follows directly as

(7) $$R_{g}=\frac{\tau_{g}d_{t}d_{r}\cos\theta_{t}\cos\theta_{r}}{\lambda }.$$

Here, $\tau_{g}$ is the threshold parameter determining the model selection; $R_{g}$ [m] is the distance threshold between the transmit and receive antenna axes; $d_{t}$ [m] and $d_{r}$ [m] are the element spacings of the transmit and receive arrays, respectively; and $\theta_{t}$ [rad] and $\theta_{r}$ [rad] are the angles between the plane defined by the incoming wavefront and the ULA axis of the transmitter and receiver, respectively.

If the transmitter–receiver separation exceeds $R_{g}$, the plane-wave approximation is sufficiently accurate with respect to the dominant eigenvalue. This condition does not imply that the channel rank is necessarily reduced to one. With a sufficient SNR and an appropriate antenna configuration, the second spatial mode may remain exploitable even beyond $R_{g}$.

**Figure 1:**
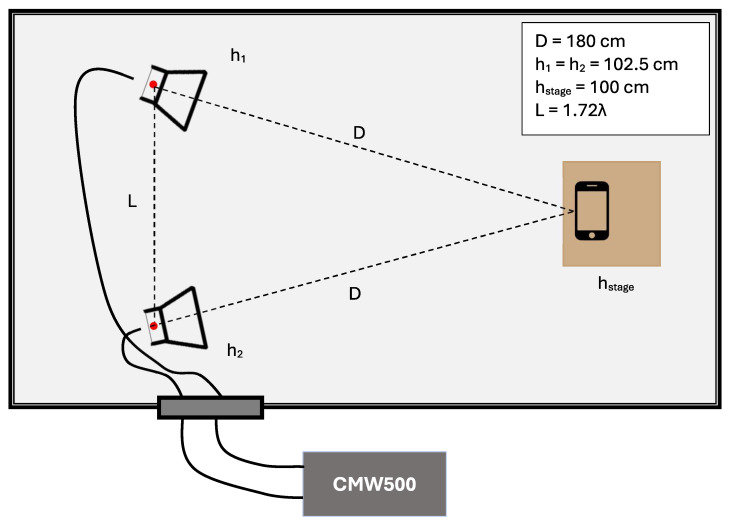
Top view of the measurement setup. The transmitting side on the left consists of two horn antennas positioned at a height of $h_{1}=h_{2}=102.5$ cm and connected to the CMW500 base-station simulator located outside the semi-anechoic chamber. The receiving side on the right consists of the user equipment (UE) placed on a non-conductive stage with a height of $h_{\mathrm{stage}}=100$ cm.

The measurement UE was rotated by 45° because, without rotation, the required UE–transmitter separation would have been about 240 cm, which would have adversely affected the link stability in the chamber. Using (7), the resulting threshold distance was $R_{g}\approx 170$ cm; therefore, separation of 180 cm was selected for the measurements. The measurement setup is shown in Figure 1.

### 3.3. Propagation Models

To estimate the cell radius corresponding to the 25 Mbit/s target data rate, two propagation models are employed. The Friis transmission equation is used as an idealized free-space upper-bound reference for frequency-dependent path loss comparison, whereas the Extended Hata model is used as an empirical terrestrial propagation estimate that accounts for additional large-scale propagation effects through correction factors. The propagation calculations do not include an additional stochastic small-scale fading component, such as a Rayleigh or Rician fading distribution. The chamber measurements represent controlled and repeatable static link-level conditions, while the propagation models are used to estimate large-scale path loss-limited cell radius values rather than fading outage probabilities.

#### 3.3.1. Free-Space Propagation (Friis Equation)

The Friis model describes propagation in an ideal, unobstructed free-space environment, where the radiated energy expands over a spherical surface and the received power decreases with the square of the distance. In this study, the model is not used as a practical network planning tool but as an upper-bound reference for comparing the relative frequency-dependent path losses of the investigated bands. The frequency dependence arises from the wavelength dependence of the effective antenna aperture [1]. The free-space attenuation term used in the Friis-based calculation is given as

(8) $$a\left[\mathrm{dB}\right]=20\cdot \log_{10}\left(\frac{\lambda }{4\pi D}\right),$$

where *D* [m] denotes the distance from the transmitting antenna and λ [m] represents the radio-signal wavelength.

#### 3.3.2. Extended Hata Model

The Extended Hata model is an empirical propagation model derived from the Hata/COST-231 model family and measurement-based path loss formulations. It generalizes the original Hata-type formulation toward a wider frequency range and transmitter–receiver separations up to 100 km. Unlike the Friis reference, this model provides a more conservative terrestrial coverage estimate by incorporating distance-dependent attenuation and correction factors associated with antenna heights and environmental conditions [40,41,42].

The fundamental equations specified in [40] have been rearranged to express the thresholds in terms of distance. The distance-dependent Equations (9)–(11) applicable to the respective frequency bands are

(9) $$\begin{array}{cc} d_{L08} & =10^{A_{08}/B},\;\mathrm{with}\\ A_{08} & =\left(L-69.6-26.2\log_{10}\left(f\right)+13.82\log_{10}\left(\max\left(30,H_{b}\right)\right)+a\left(H_{m}\right)+b\left(H_{b}\right)\right), \end{array}$$

(10) $$\begin{array}{cc} d_{L18} & =10^{A_{18}/B},\;\mathrm{with}\\ A_{18} & =\left(L-46.3-33.9\log_{10}\left(f\right)+13.82\log_{10}\left(\max\left(30,H_{b}\right)\right)+a\left(H_{m}\right)+b\left(H_{b}\right)\right), \end{array}$$

(11) $$\begin{array}{cc} d_{L21} & =10^{A_{21}/B},\;\mathrm{with}\\ A_{21} & =\left(L-46.3-10\log_{10}\left(2000^{2.39}\cdot f\right)+13.82\log_{10}\left(\max\left(30,H_{b}\right)\right)+a\left(H_{m}\right)+b\left(H_{b}\right)\right). \end{array}$$

Here, we have introduced $B=44.9-6.55\log\left(\max\left\{30;H_{b}\right\}\right)$ as the denominator of the exponents. The notation is defined as follows. The parameter *d* [km] denotes the distance between the transmitter and receiver; *f* [MHz] is the carrier center frequency; $H_{b}$ [m] is the base-station antenna height; $H_{m}$ [m] is the mobile device height; and *L* [dB] denotes the positive path loss magnitude used in the calculation. The correction factors $a\left(H_{m}\right)$ and $b\left(H_{b}\right)$ take the values 1 and 0, respectively [40].

To align the measurement results with propagation conditions characteristic of an urban environment, we derived the corresponding urban path loss values by applying the following correction to the free-space attenuation obtained during the measurements:

(12) $$\begin{array}{cc} L_{\mathrm{urban}} & =L_{\mathrm{measured}}\\ \; & \;+4.78{\left(\log_{10}\left(\min\left(\max\left(150,f\right),2000\right)\right)\right)}^{2}\\ \; & \;-18.33\log_{10}\left(\min\left(\max\left(150,f\right),2000\right)\right)+40.94, \end{array}$$

where $L_{\mathrm{measured}}$ denotes the attenuation derived from the measurement data. In this correction, the frequency argument is limited to the interval 150–2000 MHz through the nested minimum and maximum operators. Consequently, the L08 and L18 frequencies are used directly, whereas the L21 frequency of 2140 MHz is evaluated at the upper boundary of 2000 MHz in this correction term. This boundary treatment follows the applied Extended Hata formulation and may slightly reduce the frequency-dependent correction applied to the L21 case. The corrected path loss term $L_{\mathrm{urban}}$ was subsequently substituted into the distance calculation equations [40]. Accordingly, the Extended Hata results are used as empirical terrestrial coverage estimates for comparative assessment, rather than as site-specific rural network planning predictions.

### 3.4. Instrument Configuration

During the measurements, the cell output power was configured using the reference signal energy per resource element (RS EPRE) parameter, taking into account the number of resource elements. In practice, the full cell bandwidth power (FCBP) value, assuming 15 kHz subcarrier spacing, is obtained as

(13) $$\begin{array}{cc} \mathrm{FCBP}\;\left[\mathrm{dBm}\right] & =\mathrm{RS}\;\mathrm{EPRE}\;\left[\mathrm{dBm}/15\;\mathrm{kHz}\right]+10\cdot \log_{10}\left(N_{RE}\right),\\ \; & \;\;\;\;\mathrm{with}\;N_{RE}=12\cdot N_{RB}, \end{array}$$

where RS EPRE [dBm/15 kHz] denotes the reference-signal power per resource element, $N_{RE}$ is the number of resource elements, and $N_{RB}$ is the number of resource blocks [32].

By configuring the RS EPRE, the level of the downlink reference signals—and consequently the received signal level—can be directly controlled. To ensure uplink stability, the Physical Uplink Shared Channel (PUSCH) transmit power was set to its maximum value so that data transmission from the UE remained reliable under chamber attenuation conditions. In the measurement setup, the instrument-side external attenuation compensation was configured to account for the physical losses of the radiated path. The external attenuation parameter was adjusted such that effective +40 dB compensation was introduced in both the downlink and uplink signal paths. This setting ensured stable link operation despite the attenuation associated with the chamber, cables, and radiated propagation path. The applied 40 dB compensation was selected based on the calculated sectional attenuation of the measurement setup. The corresponding values for the three examined frequency bands are shown in Table 3 [12]. Here, *D* denotes the distance between the transmitting horn antennas and the UE, while $a_{0}$ corresponds to the free-space attenuation computed using (8).

Although the laboratory analysis characterizes a single controlled cell, additional outdoor measurements were performed to obtain representative band-specific effective noise-plus-interference levels under operational network conditions in the selected local environment. In the laboratory, background noise was generated using the instrument’s built-in additive white Gaussian noise (AWGN) generator. To maintain link stability during the chamber measurements, the applied AWGN level was set to −120 dBm/15 kHz in each frequency band, ensuring an SNR above 20 dB even in the 2100 MHz band at maximum transmit power. The measured outdoor values, summarized in Table 4, were then used to correct the laboratory RSRP thresholds so that the field-observed RSRP-SINR relationship could be reflected in the performance estimation in a link budget sense [43]. This correction represents the local radio noise and interference environment considered in this study and should not be interpreted as a universal band-dependent noise model.

For the MIMO configuration, open-loop spatial multiplexing (OL-SM) was employed. Preliminary closed-loop tests with PMI-based precoder adaptation introduced noticeable codeword imbalance while maintaining a comparable aggregate throughput, indicating that the instantaneous precoder selection favored one spatial mode over the other under the specific chamber realization. To improve the repeatability and reduce the dependence on instantaneous PMI selection, the reported measurements were therefore performed in open-loop mode. OL-SM uses predefined eNodeB precoding matrices without UE-specific precoder feedback, which helps to maintain more balanced two-stream excitation in the controlled radiated setup [44].

Carrier aggregation (CA) was examined in intra-band contiguous mode in the L08 band. Two 5 MHz carriers were configured, with 806 MHz selected as the primary component carrier (PCC) center frequency and 811 MHz as the secondary component carrier (SCC) center frequency, ensuring that the two carrier bandwidths did not overlap [45].

In operational networks, adaptive modulation, the precoding matrix used for spatial multiplexing, and the number of simultaneously transmitted MIMO layers are determined by the UE-reported channel quality indicator (CQI), precoding matrix indicator (PMI), and rank indicator (RI) values. Therefore, CQI-PMI-RI reporting was enabled on the base-station simulator. For CA operation, the timing of the CQI and RI reports was configured such that the CQI reports of the individual component carriers did not collide with the RI-reporting slots, as illustrated in Table 5 [32]. The analysis relies on UE-reported link adaptation indicators and measured throughput-related quantities, including the RSRP, CQI, BLER, MAC-layer throughput, and IP-layer throughput.

For IP-layer throughput evaluation, the Iperf3 tool was used via the CMW500 built-in data application unit. Data transmission was performed using the Transmission Control Protocol (TCP) in reverse mode, meaning that the server, implemented in the base-station simulator, transmitted data to the UE client. The bit rate was matched to the maximum achievable radio access network (RAN) throughput for the given measurement configuration, including the MIMO mode, CA configuration, and channel bandwidth.

Data collection was performed in continuous mode, and the measured values were aggregated using recursive averaging according to

(14) $${\bar{x}}_{n}=\frac{\left(n-1\right)\cdot {\bar{x}}_{n-1}+x_{n}}{n},$$

where *n* is the number of measurement points, ${\bar{x}}_{n-1}$ is the average of the previous n−1 measurements, and $x_{n}$ is measurement number *n* [32].

For each measurement point, the throughput was averaged over at least 60 s, while the median CQI and BLER were also recorded.

### 3.5. Measurement Method

The measurement procedure was conducted in a controlled laboratory environment using a CMW500 base-station simulator and UE configured for the investigated FR1 carrier bands. The objective was to determine throughput-related link quality thresholds for SISO and 2×2 MIMO operation in the investigated lowband and midband frequency bands and to use these thresholds as the basis for subsequent AAS and higher-order MIMO extrapolations. The procedure was divided into three phases:

1. Environmental noise characterization and system validation;
2. Throughput threshold measurement for the 25 Mbit/s target data rate;
3. Supplementary link-level measurements and extrapolations toward AAS-assisted and higher-order MIMO configurations.

#### 3.5.1. First Phase—Environmental Noise Characterization and System Validation

The measurement campaign was initiated with outdoor radio environment characterization measurements in order to obtain representative local effective noise-plus-interference conditions for the considered frequency bands. These measurements were performed during daytime working hours on the university campus, in the vicinity of one operational base-station site. The measurements were carried out as a walking test over the selected campus area, rather than at a single fixed point. During the 10 min measurement period, the QualiPoc measurement system continuously logged radio parameters, resulting in a large number of samples over the local measurement route. Therefore, the reported values represent device-reported averages over one local outdoor measurement environment. Three dedicated measurement handsets were used, each locked to one of the investigated carrier bands, namely L08, L18, or L21. Although no explicit cell lock was applied, no serving cell change was observed during the measurement period. For each band, the instantaneous RSRP and SINR values reported by the corresponding handset were recorded. The effective noise-plus-interference level was estimated from the measured RSRP-SINR relationship and normalized to 15 kHz subcarrier spacing. Accordingly, the resulting values include the receiver-observed effect of interference and ambient radio noise. The band-specific averaged values reported by the measurement system were subsequently used as correction factors to approximately preserve the outdoor RSRP-SINR relationship in the laboratory-based coverage estimation.

Apart from these noise measurements, all subsequent investigations were performed exclusively in the laboratory environment. The measurement system was then verified in the chamber under the laboratory AWGN setting used during the tests. With the base-station simulator configured to the maximum transmit power, it was confirmed that the activated cell could provide the maximum achievable data rate, corresponding to CQI = 15, to the UE in all examined frequency bands. This ensured that the selected noise generator setting and radiated link configuration did not limit the reference maximum-throughput condition used for the subsequent threshold measurements.

#### 3.5.2. Second Phase—Throughput Threshold Measurement for Coverage Estimation

For each investigated frequency band, the RSRP threshold corresponding to the 25 Mbit/s target data rate was evaluated using a 10 MHz channel bandwidth. After cell activation, Iperf-based data transmission was initiated, and the average RSRP, CQI, MAC-layer throughput, and IP-layer throughput values were recorded during 1 min measurement intervals. The transmit power was then reduced stepwise until the achieved data rate fell below the 25 Mbit/s target. In the 800 MHz band, the threshold measurement was also repeated with and without two-component-carrier aggregation in order to assess the effects of carrier aggregation under the same target rate criterion. From the resulting RSRP–throughput pairs, interpolation and, where necessary, extrapolation were used to determine the RSRP thresholds corresponding to the 25 Mbit/s target rate. The noise-corrected threshold values derived from the outdoor measurements were then used as inputs to the propagation models to estimate the cell radius of the measured 2×2 MIMO configuration. In a separate link budget step, an AAS-related beamforming gain scenario was applied to estimate the potential coverage and capacity impacts of future lowband AAS-assisted deployments. As an additional step in the 800 MHz band, the RSRP threshold corresponding to half of the 25 Mbit/s target rate was also determined, enabling conditional 4×4 MIMO extrapolation from both coverage and capacity perspectives. The parameters of the measurement cycles are summarized in Table 6.

#### 3.5.3. Third Phase—Supplementary Link-Level Measurements and Higher-Order MIMO Extrapolation

In the third phase, supplementary link-level measurements were conducted in the SISO and 2×2 MIMO configurations under different CQI conditions. The objective was to determine the maximum achievable data rate associated with each modulation and coding scheme. Since the measured throughput is affected by non-zero BLER, the maximum achievable data rate for each CQI value was estimated by scaling the measured throughput by 1/(1−BLER). For this purpose, the base-station transmit power was configured such that the measured BLER remained below 10% for the examined CQI operating point, consistent with the 3GPP CQI reporting criterion [46]. Because the primary quantity of interest in this phase was the throughput, which depends mainly on the number of resource blocks, modulation order, coding rate, and number of spatial layers, the specific frequency band used for these supplementary measurements played a secondary role. From the measured data rate ratios, the throughput enhancement associated with MIMO and carrier aggregation was derived. These results were then used to extrapolate conditional data rates for the 4×4 MIMO and 4×4 MIMO with two-component-carrier (2CC) configurations, while accounting for the associated radio-layer overhead. No physical 4×4 MIMO array or commercial AAS panel was measured in this phase. Therefore, the resulting values are treated as conditional throughput extrapolations. The configurations applied throughout the analysis are summarized in Table 7.

## 4. Measurement Results

### 4.1. Measured Lowband MIMO Performance and AAS-Assisted Scenario Extrapolation

#### 4.1.1. RSRP and Coverage

**Figure 2:**
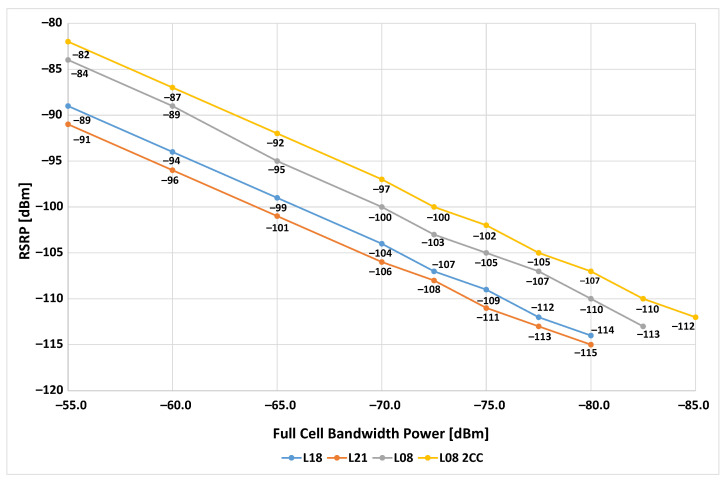
Measured RSRP as function of configured cell transmit power.

Figure 2 shows the relationship between the RSRP and the configured cell transmit power for the investigated frequency bands. As expected, reducing the transmitted reference signal power results in an approximately proportional reduction in the received RSRP. The nearly linear behavior of the curves in the logarithmic power domain confirms the stability and repeatability of the controlled radiated measurement setup. The separation between the curves reflects the frequency-dependent link attenuation of the investigated bands. For the same configured transmit power, the lower-frequency band exhibits higher received signal levels than the midband cases, which is consistent with the more favorable propagation characteristics of lowband operation. Therefore, comparing the RSRP values at identical transmit power settings provides a controlled link-level indication of the relative path loss difference between the bands. These results should be interpreted as controlled radiated link measurements rather than as direct field coverage validation. Within this scope, the measurements confirm that the experimental setup captures the expected relative received power behavior of lowband and midband links, which is subsequently used as the basis for throughput threshold and coverage estimation analysis in this work.

#### 4.1.2. Performance and Throughput

**Figure 3:**
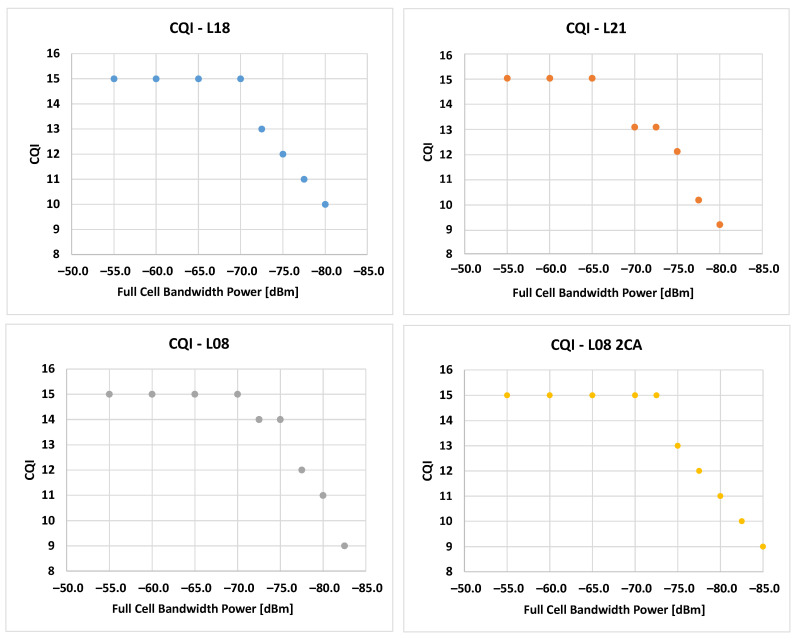
Measured CQI variation across the investigated frequency bands.

The channel quality indicator (CQI) results in Figure 3 exhibit the expected stepwise behavior. Higher received signal levels result in improved signal-to-noise conditions, enabling the use of higher-order modulation and more efficient coding schemes. As the transmit power is reduced, the reported CQI decreases in discrete steps, reflecting the transition between modulation and coding scheme (MCS) levels used by the link adaptation mechanism.

**Figure 4:**
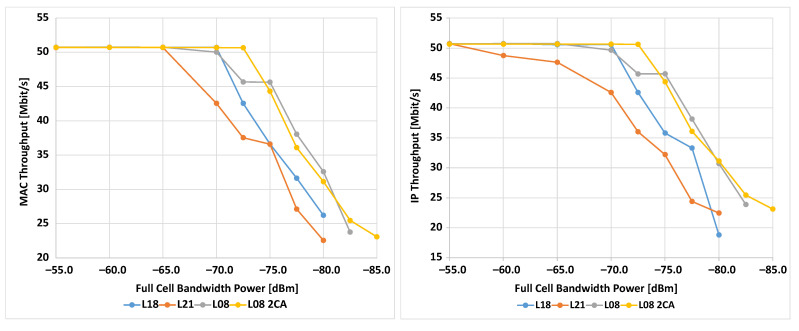
Measured MAC-layer and IP-layer throughput results.

Figure 4 presents the MAC-layer (left side) and IP-layer (right side) throughput results. The MAC throughput exhibits a plateau at high RS EPRE values, corresponding to the maximum achievable physical-layer data rate for the given configuration. As the transmit power decreases, the MAC throughput follows the CQI transitions and drops in a stepwise manner. When the block error probability increases, retransmissions and coding inefficiencies reduce the sustainable throughput.

The IP-layer throughput shows larger fluctuations than the MAC-layer throughput because transport-layer behavior is affected by retransmissions and temporary reductions in the TCP congestion window. Consequently, the IP throughput is more sensitive to short-term link degradation and provides a practical indication of the user-perceived data rate. In this study, the 25 Mbit/s threshold is therefore evaluated using the measured throughput behavior together with the CQI and BLER trends to identify the point at which stable service delivery can no longer be maintained. The BLER, rather than the bit-level error rate (BER), is used here as a system-level link stability indicator. During the threshold measurements, the CQI transitions were discrete and repeatable, and the BLER values remained stable until the link approached the throughput boundary. This behavior supported the use of the CQI, BLER, and throughput jointly in identifying the 25 Mbit/s service threshold.

To further assess the stability of the measured 2×2 MIMO baseline, the balance between the two transmitted streams was evaluated from the instrument-reported per-stream MAC throughput and CQI values. Although the measurement configuration did not provide a calibrated channel matrix from which spatial correlation or the condition number could be directly extracted, the per-stream quantities provide a link-level indication of whether one stream dominated the measured 2×2 MIMO operation. The relative stream–throughput imbalance was calculated as

(15) $$I_{\mathrm{str}}=\frac{\left|T_{1}-T_{2}\right|}{\left(T_{1}+T_{2}\right)/2}\cdot 100\%,$$

where $T_{1}$ and $T_{2}$ denote the MAC-layer throughputs of the two streams. The resulting stream balance statistics are summarized in Table 8.

The results indicate that the two-stream operation was well balanced for the L18 and L21 configurations, where the maximum per-stream MAC throughput difference was only 0.07 Mbit/s, and no CQI difference was observed between the streams. The L08 and L08 2CC cases also showed low average stream imbalance, although larger deviations appeared at the lowest received signal levels, where the link approached the throughput boundary. The maximum CQI difference remained within one CQI step in these cases. These observations do not replace direct channel correlation or eigenvalue spread measurement. However, they indicate that the selected OL-SM configuration did not rely on a strongly dominant stream, and it provided a repeatable two-stream baseline for the subsequent threshold-based extrapolations.

#### 4.1.3. Coverage Boundary Determination for the 25 Mbit/s Target Rate

The linear interpolation formula between two adjacent measurement points is given by

(16) $$x^{*}=x_{1}+\left(y^{*}-y_{1}\right)\cdot \frac{x_{2}-x_{1}}{y_{2}-y_{1}},$$

where $\left(x_{1},y_{1}\right)$ and $\left(x_{2},y_{2}\right)$ denote two adjacent measurement points—for example, the RSRP and throughput pairs. In the present case, $y^{*}=25\;\mathrm{Mbit}/s$ is the target throughput, while $x^{*}$ denotes the corresponding RSRP threshold. The interpolation was applied only in the local transition region around the target throughput, where the throughput changed monotonically with the RSRP. Points on the high-throughput plateau were not used for threshold extraction, because small differences between $y_{1}$ and $y_{2}$ would make the interpolation ill-conditioned and could amplify measurement errors in the estimated RSRP threshold. If the target value fell outside the directly measured interval, extrapolation was performed using the same expression based on the nearest boundary points. The selected threshold was interpreted together with the observed CQI and BLER behavior, and the resulting coverage sensitivity is further quantified through the effective noise-plus-interference uncertainty analysis reported below.

The reference noise level was set to −120 dBm/15 kHz during the chamber measurements. Under this laboratory noise condition, the interpolated RSRP thresholds required to sustain the 25 Mbit/s target rate were first determined from the measured RSRP–throughput curves. Since the outdoor measurements indicated higher and band-dependent effective noise-plus-interference levels, a band-specific correction was subsequently applied to the laboratory RSRP thresholds. The correction was calculated as

(17) $$\Delta N_{b}=N_{\mathrm{out},b}-N_{\mathrm{lab}},$$

where *b* denotes the frequency band, $N_{\mathrm{out},b}$ is the measured outdoor effective noise-plus-interference level, and $N_{\mathrm{lab}}=-120$ dBm/15 kHz is the laboratory reference noise level. The corrected RSRP threshold was then obtained as

(18) $${\mathrm{RSRP}}_{\mathrm{corr},b}={\mathrm{RSRP}}_{\mathrm{uncorr},b}+\Delta N_{b}.$$

The purpose of this correction was to preserve, in an approximate link budget sense, the SINR condition associated with the measured throughput threshold. For a fixed bandwidth, modulation and coding behavior, and target throughput, an increase in the effective noise-plus-interference level requires a corresponding increase in received signal power to maintain a comparable SINR. Therefore, the corrected RSRP thresholds should be interpreted as scenario-specific thresholds representing the measured local radio environment, rather than as universal receiver sensitivity values. Table 9 summarizes both the uncorrected laboratory thresholds and the resulting band-specific noise-corrected thresholds used for the subsequent coverage estimation.

For the cell radius estimates, a base-station antenna height of $H_{b}=35$ m, a user equipment height of $H_{m}=1.5$ m, and an EIRP of 46 dBm were assumed. These values represent a typical macro-cell-oriented downlink link budget configuration used to obtain comparative radius estimates under identical assumptions across the investigated bands. Since the Extended Hata distance exponent depends on $H_{b}$, the resulting radii should be interpreted as comparative scenario estimates for this selected antenna height configuration, rather than as site-specific planning results. The resulting radii are summarized in Table 10.

Because the coverage estimates depend directly on the band-specific effective noise-plus-interference correction, a sensitivity analysis was performed for the Extended Hata cell radius estimates. The uncertainty parameter $\Delta N_{\mathrm{sens}}$ represents an additional deviation in the effective outdoor noise-plus-interference level from the baseline value used in Table 9. For the Extended Hata model, the radius scaling can be expressed as

(19) $$d\left(\Delta N_{\mathrm{sens}}\right)=d_{0}\cdot 10^{-\Delta N_{\mathrm{sens}}/B},$$

where $d_{0}$ is the baseline Extended Hata radius and $B=44.9-6.55\log_{10}\left(\max\left(30,H_{b}\right)\right)$. The resulting sensitivity values for the measured 2×2 MIMO case are summarized in Table 11.

The sensitivity analysis shows that ±3 dB uncertainty in the effective noise-plus-interference correction changes the Extended Hata radius estimates by +22% and −18%, respectively, while ±5 dB uncertainty changes them by +39% and −28% after rounding. Since the same correction mechanism affects the allowable path loss magnitude in the AAS-assisted and conditional 4×4 MIMO cases, the relative Extended Hata radius scaling is the same for these scenarios when the same base-station height is used. Therefore, the sensitivity table is reported for the measured 2×2 MIMO baseline, while the same proportional effect should be considered when interpreting the extrapolated coverage scenarios.

To assess the potential impacts of future lowband AAS deployments, a separate AAS-assisted link budget scenario was evaluated by adding a representative 10 dB AAS-related beamforming gain parameter to the assumed downlink EIRP. In this study, this value is interpreted as an EIRP-equivalent link budget margin in the direction of the served UE, representing the combined effect of the directional array gain and beamforming toward the target link. It is not a measured gain of the experimental horn antenna arrangement and should not be interpreted as a guaranteed system-level SINR gain in a deployed AAS network. In practical networks, SINR improvements may also include interference suppression, scheduling effects, and multi-user precoding gains, which are outside the scope of the present single-cell link budget calculation. The selected 10 dB value is therefore used as a moderate representative scenario parameter and is consistent with cell-edge beamforming improvements reported for AAS and optimized precoding in the literature [25,26].

The same 10 dB parameter was applied to all investigated bands to provide a common comparative scenario for evaluating frequency-dependent propagation effects under an identical link budget improvement. However, the physical realization of a given beamforming gain is frequency-dependent, since the achievable gain is related to the effective antenna aperture relative to the wavelength. Therefore, obtaining the same directional gain at 800 MHz generally requires a larger physical aperture than at 1800 or 2100 MHz, and practical band-specific AAS implementations require antenna pattern, aperture, and deployment-specific validation. For the Extended Hata configuration used in this study, the 10 dB link budget margin corresponds to a 94% radius increase after rounding for a fixed base-station height. If smaller realizable AAS-related margins were assumed, the radius increase would decrease accordingly; for example, 4 dB, 6 dB, and 8 dB margins would correspond to 30%, 49%, and 70% Extended Hata radius increases after rounding, respectively. For comparison, under the Friis upper-bound reference, 4 dB, 6 dB, 8 dB, and 10 dB link budget margins correspond to 58%, 100%, 151%, and 216% radius increases after rounding, respectively. The corresponding scenario-based estimates for the 10 dB case are shown in Table 12.

**Figure 5:**
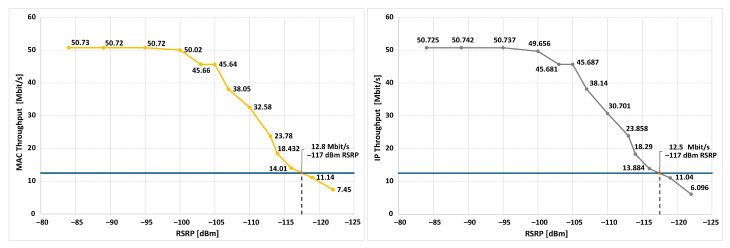
Measured 2×2 MIMO MAC- and IP-layer throughputs as a function of RSRP. The horizontal line marks the 12.5 Mbit/s half-rate threshold, and the vertical dashed line indicates the selected −117 dBm RSRP threshold.

To obtain a conditional 4×4 MIMO cell radius extrapolation for the 25 Mbit/s target rate, the required RSRP threshold was inferred from the measured 2×2 MIMO throughput behavior. Specifically, the 4×4 MIMO case was approximated by identifying the RSRP level at which the measured 2×2 MIMO link approached half of the target rate, i.e., 12.5 Mbit/s. For this purpose, an additional 2×2 MIMO threshold sweep was evaluated using both the MAC-layer and IP-layer throughput, as shown in Figure 5. The measured curves indicate that the half-rate condition is reached near −117 dBm RSRP. At this point, the MAC-layer throughput is about 12.8 Mbit/s, while the IP-layer throughput is close to 12.5 Mbit/s. Therefore, the laboratory RSRP threshold for the conditional 4×4 MIMO extrapolation was set to −117 dBm. This approach represents an ideal four-layer scaling assumption. If the available number of effective spatial layers is doubled from two to four and the additional layers provide comparable quality, the same aggregate target rate can be sustained at a lower per-layer throughput requirement. Under the common laboratory noise setting of −120 dBm/15 kHz, the corresponding RSRP threshold was treated as band-independent before applying the band-specific noise corrections. The resulting cell radius values therefore represent conditional upper-bound coverage estimates. In practical lowband deployments, spatial correlation, rank deficiency, layer imbalance, and the propagation conditions may reduce the effective 4×4 MIMO gain. In such cases, the achievable cell radius would be lower than the ideal conditional estimate reported here.

Table 13 summarizes the resulting RSRP thresholds after applying the band-specific noise corrections. Table 14 provides the corresponding conditional cell radius estimates for the 56 dBm EIRP AAS-assisted link budget scenario.

### 4.2. Conditional Throughput Extrapolation Toward 4×4 MIMO and Carrier Aggregation

The available two transmitting antennas allowed the direct measurement of the SISO, 2×2 MIMO, and 2×2 MIMO with two-component-carrier aggregation configurations. The expected throughput of the 4×4 MIMO configurations was therefore estimated by extrapolating the measured data rate relationships. Table 15 compares the measured MAC-layer throughputs across different CQI levels. For each configuration, the maximum observed throughput value was used as the basis for extrapolation.

The measured throughput ratios in Table 15 follow the expected scaling with bandwidth and spatial layers. Doubling the bandwidth from 10 MHz to 20 MHz nearly doubled the throughput, with a small 0.5–1% surplus caused by the discrete transport block size (TBS) values defined in the 3GPP tables, as summarized in Table 16. The 10 MHz SISO and 2×2 MIMO comparison showed an almost twofold gain at CQI = 15, while lower CQI values resulted in a ratio close to 1.9. The overhead correction factors for MIMO and carrier aggregation were therefore derived from the maximum throughput values at CQI = 15, where the instrument-reported values are most stable. These correction factors therefore characterize high-CQI, maximum-throughput operation and are used as overhead adjustment factors in the throughput projection, not as measured cell-edge MIMO efficiency values. For other CQI values, the lower throughput margins make the overhead fraction more sensitive to measurement variability.

The maximum throughput values for the 4×4 MIMO and 4×4 MIMO 2CC configurations were extrapolated using 3GPP TS 36.213 and the measured throughput ratios [46]. The relevant TBS tables define the number of transmitted bits for different bandwidths and spatial-layer configurations. These tables were used to derive the four-layer TBS values from the measured SISO and 2×2 MIMO cases. For CQI values below 15, the CMW500 CQI table was used to scale the maximum RAN throughput according to the corresponding modulation and coding rate. Since the TBS represents the number of bits transmitted per 1 ms transmission time interval (TTI), the resulting value was converted to Mbit/s and multiplied by 0.8 to account for the scheduler configuration, in which 8 out of 10 subframes carry user data. The resulting values were corrected using the overhead factors derived from the measurements. For 4×4 MIMO, the square of the measured MIMO correction factor, 0.9943, was applied to represent two successive layer-doubling steps. For 2CC operation, an additional carrier aggregation correction factor of 0.9992 was used. The final extrapolated throughput values are summarized in Table 17. These values assume that four spatial layers can be supported with sufficient channel rank and decorrelation. Consequently, they should be interpreted as conditional throughput estimates rather than guaranteed cell-edge user rates.

Expressed as the effective MAC-layer spectral efficiency, the extrapolated 10 MHz 4×4 MIMO case at CQI = 15 corresponds to about 10.16 bit/s/Hz, while the 5 MHz 2CC case yields a comparable effective bandwidth-normalized throughput. These values represent throughput-based effective efficiencies after applying the assumptions and overhead corrections described above. The values in Table 17 should be interpreted as ideal high-CQI four-layer TBS-based throughput projections when sufficient rank and layer balance are available. In practical lowband deployments, the effective four-layer gain may be reduced by spatial correlation, rank deficiency, and layer imbalance. As an illustrative sensitivity example, if the effective four-layer efficiency were reduced to 75% of the ideal value, the CQI = 15 throughput would decrease from 101.628 Mbit/s to about 76.2 Mbit/s in the 10 MHz case and from 203.094 Mbit/s to about 152.3 Mbit/s in the 10 MHz 2CC case. With a more conservative 50% effective four-layer efficiency, the corresponding values would be about 50.8 Mbit/s and 101.5 Mbit/s, respectively. Therefore, the 4×4 MIMO throughput values should be interpreted as conditional high-CQI projections rather than guaranteed deployment performance.

### 4.3. Summary of Coverage and Capacity Results

The measurement results confirm the expected frequency-dependent received power behavior under controlled radiated conditions. For identical configured transmit power levels, the lowband link provided higher received signal levels and more favorable CQI values than the midband links. These measured RSRP–throughput thresholds were then used for a coverage estimate comparison under identical throughput requirements. For the measured 2×2 MIMO case, the 800 MHz band provided a larger estimated cell radius for the 25 Mbit/s target rate than the 1800 MHz band under the applied propagation model assumptions. The difference was 84% under the Friis upper-bound reference and 43% under the Extended Hata model. The Extended Hata value is emphasized because it provides the more conservative terrestrial estimate. The corrected results remain scenario-specific, since the outdoor measurements indicated band-dependent effective noise-plus-interference levels in the considered local radio environment. The sensitivity analysis shows that, under the Extended Hata model, ±3 dB uncertainty in this correction changes the estimated radii by approximately +22% and −18%, while ±5 dB uncertainty changes them by approximately +39% and −28%.

The AAS-assisted link budget scenario increased the estimated cell radius in all investigated bands. The increase was 216% under the Friis upper-bound reference and 93% under the Extended Hata model. These values represent scenario-based coverage extensions associated with the assumed 10 dB EIRP-equivalent AAS-related beamforming gain parameter, not a measured AAS gain or guaranteed system-level SINR improvement.

The 800 MHz coverage advantage over the 2100 MHz band was smaller than its advantage over the 1800 MHz band. This is explained by the roughly 5 dB lower measured noise level in the 2100 MHz band. Under the Extended Hata model, the corresponding 800 MHz coverage advantage over 2100 MHz was 13%, both with and without the AAS-assisted link budget scenario. This indicates that the estimated cell radius is influenced not only by the carrier frequency but also by the measured local noise and interference environment used in the correction.

The conditional 4×4 MIMO extrapolation further increased the estimated 800 MHz coverage advantage. Under the Friis upper-bound reference, the 800 MHz radius exceeded the 1800 MHz and 2100 MHz values by 123% and 42%, respectively. Under the Extended Hata model, the corresponding increases were 59% and 20%. These values should be interpreted together with the rank and decorrelation assumptions described above.

The throughput extrapolation indicates that conditional 4×4 MIMO operation can exceed 100 Mbit/s in a 10 MHz bandwidth under ideal high-CQI four-layer assumptions, while the combination of 4×4 MIMO and two-component-carrier aggregation can exceed 200 Mbit/s in the corresponding ideal case. For a 5 MHz bandwidth, the 25 Mbit/s target rate remains achievable over a broad CQI range in the extrapolated table, while 100 Mbit/s requires the combined use of 4×4 MIMO and two-component-carrier aggregation. Based on the CQI-dependent extrapolated values, all examined configurations except the 5 MHz 4×4 MIMO case can sustain the 25 Mbit/s target rate down to CQI = 7. The illustrative non-ideal four-layer sensitivity example shows that these high-CQI throughput values decrease when the effective layer efficiency is reduced, and therefore the reported 4×4 MIMO throughput values should be interpreted as conditional projections.

## 5. Conclusions and Future Work

This study evaluated the coverage and capacity potential of lowband MIMO links under a fixed 25 Mbit/s downlink service target. The experimental part was based on controlled radiated SISO and 2×2 MIMO measurements, while the potential impacts of the AAS-related beamforming gain, 4×4 MIMO, and carrier aggregation were evaluated through clearly defined extrapolation steps. The measured 2×2 MIMO thresholds led to a larger estimated coverage radius for the 800 MHz band than for the 1800 MHz band at the same target data rate. The relative increase was 84% under the Friis upper-bound reference and 43% under the Extended Hata model.

Building on the measured 2×2 MIMO thresholds, the AAS-assisted link budget scenario and the higher-order MIMO extrapolations indicate how the limited bandwidth of lowband operation can be partly compensated for by the beamforming gain and improved spatial efficiency. When the representative 10 dB EIRP-equivalent AAS-related beamforming gain scenario was applied, the estimated cell radius increased by 216% under the Friis upper-bound reference and by 93% under the Extended Hata model. With the conditional 4×4 MIMO extrapolation, the estimated 800 MHz coverage advantage over the 1800 MHz band increased to 123% under the Friis upper-bound reference and to 59% under the Extended Hata model. These values indicate that, if sufficient channel rank and spatial decorrelation are available, higher-order lowband MIMO can extend the area in which the 25 Mbit/s target rate is sustainable. Moreover, when additional spatial layers are combined with AAS-related beamforming gains, the resulting improvement in radio link quality and effective spectral efficiency can enhance the service stability and achievable data rate at already covered cell-edge locations.

The throughput extrapolations further support this interpretation. In the considered ideal high-CQI four-layer configuration, 4×4 MIMO is estimated to exceed 100 Mbit/s in a 10 MHz bandwidth, while 4×4 MIMO with two-component-carrier aggregation is estimated to exceed 200 Mbit/s. The illustrative non-ideal four-layer sensitivity example shows that these throughput values decrease with reduced effective layer efficiency, confirming that the 4×4 MIMO projections should be interpreted as conditional rather than guaranteed performance levels. Thus, although the lowband spectrum remains inherently bandwidth-limited compared with midband and higher-frequency allocations, AAS operation, higher-order MIMO, and carrier aggregation can substantially improve its service capabilities. This suggests that the lowband layer can provide a stable and robust service layer for general users, moderate-throughput applications, rural coverage, and indoor or penetration-limited scenarios.

Taken together, the results suggest that future lowband AAS deployments could support the evolution of the lowband layer from a mainly coverage-oriented layer toward a more capable coverage-and-capacity support layer, where the main benefit is improved service robustness rather than peak rate maximization. The main value of this deployment concept is the combination of favorable propagation, improved building penetration, beamforming gains, and increased spatial-layer support. This combination can improve service continuity at the cell edge and reduce the need to serve all general traffic through midband or higher-frequency resources. As a result, higher bands can be reserved for services requiring larger bandwidths or higher peak rates or those with more specific quality-of-service requirements, while the lowband layer maintains reliable baseline connectivity over a larger and more robust service area.

The results should be interpreted within the assumptions of the present measurement and extrapolation framework, with a clear distinction between the directly measured SISO and 2×2 MIMO thresholds and the extrapolated AAS-assisted and higher-order MIMO scenarios. The experimental setup directly measured SISO and 2×2 MIMO links using passive horn antennas, and the coverage estimates depend on the local effective noise-plus-interference correction applied to the laboratory thresholds. The dominant measurement-related uncertainty in the coverage estimation is therefore associated with the effective noise-plus-interference correction and the RSRP threshold selection around the target throughput boundary. The sensitivity analysis quantifies the impact of this uncertainty on the Extended Hata radii, showing that a ±3 dB deviation changes the estimated radii by +22% and −18%, while a ±5 dB deviation changes them by +39% and −28% after rounding. The added per-stream analysis confirms balanced two-stream operation in the selected OL-SM configuration, but direct channel correlation and eigenvalue spread measurements remain necessary for full spatial rank characterization. The AAS-related gain was evaluated as an EIRP-equivalent link budget scenario parameter, while the 4×4 MIMO performance was evaluated through conditional coverage and throughput extrapolations. Therefore, the achievable gain in a deployed network will depend on the actual AAS radiation pattern, channel rank, scheduler behavior, inter-cell interference, and applied precoding strategy [7]. Furthermore, the present analysis is downlink-oriented. In practical rural and penetration-limited deployments, uplink coverage may become the limiting direction because of UE transmit power constraints. Therefore, the downlink coverage extensions reported here do not by themselves guarantee symmetric service availability. These factors define the next validation steps.

Future work will therefore focus on extending the present link-level framework toward direct system-level validation. The purpose of these next steps is to verify how the lowband MIMO and AAS-assisted gains estimated in this study can be realized under practical deployment conditions. In particular, the following research directions are considered essential:

- Direct lowband 4×4 MIMO measurements, including rank indicator statistics and channel rank characterization, to verify whether the extrapolated spatial-layer gains are achievable;
- Outdoor validation measurements in rural, suburban, and indoor-to-outdoor scenarios to quantify how the laboratory-derived thresholds translate into practical coverage performance;
- Measurements or emulation with representative lowband AAS radiation patterns to evaluate realistic beamforming gain, beamwidth, side-lobe behavior, and polarization effects;
- Multi-user and multi-cell evaluations to assess scheduler behavior, inter-cell interference, and the practical efficiency of beam-based spatial reuse;
- Uplink coverage analysis, since the limited transmit power of user equipment may constrain the practical lowband cell radius even when downlink coverage is enhanced by AAS operation.

## Figures and Tables

**Table 1:** Equipment used in the measurements [31,32,33,34].

Name	Type	Manufacturer
RF Cable (2)	NN20	Rosenberg Micro Coax (Rosenberger Hochfrequenztechnik GmbH & Co. KG, Fridolfing, Germany)
RF Cable (2)	NN30	Rosenberg Micro Coax (Rosenberger Hochfrequenztechnik GmbH & Co. KG, Fridolfing, Germany)
Measurement Cell Phone	Galaxy S21+ 5G	Samsung Qualipoc (Rohde & Schwarz GmbH & Co. KG, München, Germany)
Horn Antenna (2)	Model 3115	TS-Lindgren (ETS-Lindgren, Cedar Park, TX, USA)
Base Station Simulator	CMW500	Rohde & Schwarz (Rohde & Schwarz GmbH & Co. KG, München, Germany)
Test SIM-Card	R&S CMW	Rohde & Schwarz (Rohde & Schwarz GmbH & Co. KG, München, Germany)

**Table 2:** Antenna spacing used for the tested mobile frequency bands.

Parameter	L21 Band 1	L18 Band 3	L08 Band 20
Downlink center frequency *f* [MHz]	2140	1835	806
Wavelength λ [m]	0.1402	0.1635	0.3722
Antenna spacing *d* [m]	0.24	0.28	0.64

Antenna spacing corresponds to approximately 1.72λ for each tested band.

**Table 3:** Link budget terms of the measurement setup.

B	λ [m]	*D* [m]	$a_{0}$ [dB]	$L_{C}$ [dB]	$G_{H}$ [dBi]	$G_{\mathrm{Ph}}$ [dBi]	$L_{P}$ [dB]
1	0.1402	1.8	−44.154	2.24	4.8	1.0	−40.594
3	0.1635	1.8	−42.819	2.11	4.6	1.0	−39.329
20	0.3722	1.8	−35.674	2.00	1.8	1.0	−34.874

B: band; $a_{0}$: free-space attenuation for distance *D*, computed from the Friis equation, with the subsequent cable loss $L_{C}$, horn antenna gain $G_{H}$, and phone antenna gain $G_{Ph}$ producing the net link budget term $L_{P}$.

**Table 4:** Measured average outdoor effective noise-plus-interference level at 15 kHz bandwidth across the three frequency bands.

Quantity	L21	L18	L08
Noise level [dBm/15 kHz]	−96.4845	−91.2049	−91.0380

The values represent band-specific device-reported averages obtained from 10 min QualiPoc outdoor walking measurements on the selected university campus route. They characterize the effective receiver-observed noise-plus-interference level in the local radio environment and are not intended as universal band-dependent noise values.

**Table 5:** Correct and incorrect RI (R) and CQI (C) report configuration across LTE subframes for primary (PCC) and secondary (SCC) component carriers [32].

Configuration	Subframe	0	1	2	3	4	5	6	7	8	9
Incorrect	PCC	R	C				R	C			
Incorrect	SCC		R	C				R	C		
Correct	PCC	R	C				R	C			
Correct	SCC			R	C				R	C	

**Table 6:** Parameters of the measurement cycles—second phase.

MIMO/SISO	Band	*f* [MHz]	BW [MHz]	CA	FCBP [dBm]
2×2 MIMO	Band 1	2140.0	10	N/A	−55.0→−80.0
2×2 MIMO	Band 3	1835.0	10	N/A	−55.0→−80.0
2×2 MIMO	Band 20	806.0	10	N/A	−55.0→−82.5
2×2 MIMO	Band 20	808.5	5	2CC	−55.0→−82.5

*f*: center frequency; FCBP: full cell bandwidth power, representing the configured downlink cell power during throughput threshold testing; N/A: not applicable, indicating that carrier aggregation was not applied; 2CC: two-component-carrier aggregation.

**Table 7:** Parameters of the measurement cycles—third phase.

MIMO/SISO	Band	*f* [MHz]	BW [MHz]	CA	CQI
1×1 SISO	Band 3	1835	10	N/A	15→13
1×1 SISO	Band 3	1835	20	N/A	15→13
2×2 MIMO	Band 3	1835	10	N/A	15→13
2×2 MIMO	Band 20	808.5	5	2CC	15→13

*f*: center frequency; CQI values were evaluated under controlled RS EPRE conditions to determine the achievable modulation and coding scheme for the corresponding link configuration; N/A: not applicable, indicating that carrier aggregation was not applied; 2CC: two-component-carrier aggregation.

**Table 8:** Per-stream balance statistics for the measured 2×2 MIMO configurations.

Configuration	Valid Points	Max. Diff. [Mbit/s]	Mean Diff. [Mbit/s]	Max. Imbalance [%]	Max. CQI Diff.
L18 2×2 MIMO	8	0.07	0.009	0.33	0
L21 2×2 MIMO	8	0.07	0.010	0.33	0
L08 2×2 MIMO	9	1.50	0.170	12.62	1
L08 2×2 MIMO 2CC	10	0.49	0.113	3.15	1

The stream difference is calculated from the absolute difference between the instrument-reported per-stream MAC throughputs. The relative imbalance is calculated using (15). The maximum CQI difference denotes the largest observed CQI difference between the two streams over the evaluated transmit power points.

**Table 9:** Uncorrected and noise-corrected RSRP thresholds for the 25 Mbit/s target rate.

Quantity	L21	L18	L08	L08 and 2CC
Uncorrected RSRP [dBm]	−113.51	−114.25	−112.58	−110.39
Noise-corrected RSRP [dBm]	−89.99	−85.45	−83.62	−81.43

The uncorrected values correspond to the laboratory RSRP thresholds obtained under the −120 dBm/15 kHz reference noise setting. The noise-corrected values were obtained by adding the measured band-specific outdoor effective noise-plus-interference differences to the corresponding laboratory thresholds and were used as inputs for the subsequent coverage estimation.

**Table 10:** Estimated cell radius for the 25 Mbit/s target rate at 46 dBm EIRP.

Band	Center Frequency [MHz]	Path Loss [dB]	Radius (Friis) [m]	Radius (Extended Hata) [m]
L21	2140	108.21	2870	1271
L18	1835	103.67	1985	1010
L08	806	101.84	3660	1443
L08 and 2CC	808.5	99.65	2836	1246

The path loss magnitudes are positive values used in the Friis and Extended Hata calculations.

**Table 11:** Sensitivity of the Extended Hata cell radius estimates to the effective noise-plus-interference uncertainty for the measured 2×2 MIMO case.

Band	$\Delta N_{\mathrm{sens}}=-5\;\mathrm{dB}$	$\Delta N_{\mathrm{sens}}=-3\;\mathrm{dB}$	Baseline	$\Delta N_{\mathrm{sens}}=+3\;\mathrm{dB}$	$\Delta N_{\mathrm{sens}}=+5\;\mathrm{dB}$
L21	1770	1550	1271	1042	913
L18	1406	1232	1010	828	725
L08	2009	1760	1443	1183	1036
L08 and 2CC	1735	1520	1246	1022	895

All values are cell radius estimates in meters. Negative $\Delta N_{\mathrm{sens}}$ values represent a lower effective noise-plus-interference level than the baseline correction, resulting in larger radii. Positive values represent a higher effective noise-plus-interference level, resulting in smaller radii.

**Table 12:** AAS-assisted EIRP-equivalent link budget scenario for the 25 Mbit/s target rate at 56 dBm effective EIRP.

Band	Center Frequency [MHz]	Path Loss [dB]	Radius (Friis) [m]	Radius (Extended Hata) [m]
L21	2140	118.21	9077	2463
L18	1835	113.67	6276	1957
L08	806	111.84	11574	2796
L08 and 2CC	808.5	109.65	8967	2415

The path loss magnitudes include the additional 10 dB EIRP-equivalent link budget margin associated with the assumed AAS-related beamforming gain scenario. The value is a scenario parameter and does not represent a measured AAS gain.

**Table 13:** Noise-corrected RSRP thresholds for the conditional 4×4 MIMO cell radius extrapolation at the 25 Mbit/s target rate.

Quantity	L21	L18	L08	L08 and 2CC
RSRP corrected [dBm] (4×4 MIMO)	−93.48	−88.20	−88.03	−88.03

The values represent estimated downlink RSRP thresholds required to sustain the 25 Mbit/s target rate under the conditional 4×4 MIMO extrapolation based on measured 2×2 MIMO chamber data.

**Table 14:** Conditional 4×4 MIMO cell radius extrapolation for the 25 Mbit/s target rate at 56 dBm effective EIRP.

Band	Center Frequency [MHz]	Path Loss [dB]	Radius (Friis) [m]	Radius (Extended Hata) [m]
L21	2140	121.70	13565	3103
L18	1835	116.42	8614	2348
L08	806	116.25	19231	3744
L08 and 2CC	808.5	116.25	19171	3739

The radii correspond to conditional coverage estimates for achieving the 25 Mbit/s downlink target rate under 4×4 MIMO with an assumed 10 dB EIRP-equivalent AAS-related beamforming gain parameter.

**Table 15:** Throughput results for different CQI values and throughput ratios.

**Configuration**	**CQI 15**	**CQI 13**	**CQI 11**
L18 SISO 10 MHz [Mbit/s]	25.363	23.038	17.056
L18 SISO 20 MHz [Mbit/s]	51.021	47.573	34.214
L18 2×2 MIMO 10 MHz [Mbit/s]	50.730	43.800	32.602
L08 2×2 MIMO 5 MHz 2CC [Mbit/s]	50.690	46.940	32.400
**Configuration**	**Throughput ratio**		
10 MHz SISO/20 MHz SISO	2.0116	2.0650	2.0060
10 MHz SISO/10 MHz 2×2 MIMO	2.0002	1.9012	1.9115
MIMO overhead loss–L18 20 MHz SISO/L18 10 MHz 2×2 MIMO	0.9943	–	–
CA overhead loss–L18 10 MHz 2×2 MIMO/L08 2×2 MIMO 5 MHz 2CC	0.9992	–	–

**Table 16:** Transport block size values used for the bandwidth and spatial-layer throughput projection.

Bandwidth	Number of RBs	Max. TBS, 1 Layer	Max. TBS, 2 Layers	Max. TBS, 4 Layers
5 MHz	25	15,840	31,704	63,776
10 MHz	50	31,704	63,776	128,496
20 MHz	100	63,776	128,496	254,328

The TBS values correspond to the maximum transport block sizes used in the throughput projection. They illustrate the discrete scaling behavior with the bandwidth and number of spatial layers.

**Table 17:** Throughput extrapolation for different 4×4 MIMO bandwidth and carrier aggregation configurations. The values were obtained using four-layer TBS extrapolation and corrected with the measured MIMO and CA overhead factors.

CQI	10 MHz [Mbit/s]	10 MHz 2CC [Mbit/s]	5 MHz [Mbit/s]	5 MHz 2CC [Mbit/s]
15	101.628	203.094	50.441	100.801
14	98.160	196.162	48.719	97.361
13	91.338	182.530	45.334	90.595
12	76.770	153.418	38.103	76.145
11	65.787	131.468	32.652	65.251
10	54.803	109.518	27.200	54.357
9	46.556	93.037	23.107	46.177
8	35.610	71.164	17.674	35.320
7	28.750	57.455	14.270	28.516
6	22.391	44.747	11.113	22.209
5	15.763	31.500	7.823	15.634
4	10.290	20.564	5.107	10.206
3	7.978	15.943	3.960	7.913
2	5.010	10.012	2.487	4.969
1	5.010	10.012	2.487	4.969

## Data Availability

The original contributions presented in this study are included in the article. Further inquiries can be directed to the corresponding author.

## Abbreviations

The following abbreviations are used in this manuscript:

2CC	Two-Component Carrier
3GPP	Third-Generation Partnership Project
4G LTE	Fourth Generation of Wireless Cellular Technology—Long-Term Evolution
5G NR	Fifth Generation of Wireless Cellular Technology—New Radio
6G	Sixth Generation of Wireless Cellular Technology
AAS	Active Antenna System
ARA	Agriculture and Rural Communities
AWGN	Additive White Gaussian Noise
BER	Bit Error Rate
BLER	Block Error Rate
BW	Bandwidth
CA	Carrier Aggregation
CC	Component Carrier
CQI	Channel Quality Indicator
EIRP	Equivalent Isotropically Radiated Power
eNodeB	Evolved Node B
FCBP	Full Cell Bandwidth Power
FR1	Frequency Range 1
FWA	Fixed Wireless Access
IP	Internet Protocol
LOS	Line-of-Sight
MAC	Medium Access Control
MCS	Modulation and Coding Scheme
MIMO	Multiple-Input Multiple-Output
MMSE	Minimum Mean-Square Error
MU–MIMO	Multi-User Multiple-Input Multiple-Output
NTN	Non-Terrestrial Network
OL-SM	Open-Loop Spatial Multiplexing
PCC	Primary Component Carrier
PMI	Precoding Matrix Indicator
PUSCH	Physical Uplink Shared Channel
PWM	Plane-Wave Model
RAN	Radio Access Network
RB	Resource Block
RF	Radio Frequency
RI	Rank Indicator
RS EPRE	Reference Signal Energy per Resource Element
RSRP	Reference Signal Received Power
SAC	Semi-Anechoic Chamber
SCC	Secondary Component Carrier
SIM	Subscriber Identification Module
SINR	Signal-to-Interference-plus-Noise Ratio
SISO	Single-Input Single-Output
SNR	Signal-to-Noise Ratio
SVD	Singular Value Decomposition
SWM	Spherical-Wave Model
TBS	Transport Block Size
TCP	Transmission Control Protocol
TN	Terrestrial Network
TS	Technical Specification
TTI	Transmission Time Interval
UE	User Equipment
ULA	Uniform Linear Array
ZF	Zero-Forcing
